# New thermal decomposition pathway for TATB

**DOI:** 10.1038/s41598-023-47952-6

**Published:** 2023-12-01

**Authors:** Keith D. Morrison, Ana Racoveanu, Jason S. Moore, Alan K. Burnham, Batikan Koroglu, Keith R. Coffee, Adele F. Panasci-Nott, Gregory L. Klunder, Bradley A. Steele, M. A. McClelland, John G. Reynolds

**Affiliations:** 1 Lawrence Livermore National Laboratory, Nuclear and Chemical Sciences Division, Livermore, CA 94550 USA; 2 NorthWind Services LLC, Livermore, CA 94550 USA; 3 Lawrence Livermore National Laboratory, Materials Science Division, Livermore, CA 94550 USA; 4 Stratify//MH Chew Associates, Livermore, CA 94551 USA

**Keywords:** Analytical chemistry, Chemical engineering, Chemical safety, Energy, Materials chemistry, Organic chemistry, Process chemistry, Chemical synthesis, Theoretical chemistry

## Abstract

Understanding the thermal decomposition behavior of TATB (1,3,5-triamino-2,4,6-trinitrobenzene) is a major focus in energetic materials research because of safety issues. Previous research and modelling efforts have suggested benzo-monofurazan condensation producing H_2_O is the initiating decomposition step. However, early evolving CO_2_ (m/z 44) along with H_2_O (m/z 18) evolution have been observed by mass spectrometric monitoring of head-space gases in both constant heating rate and isothermal decomposition studies. The source of the CO_2_ has not been explained, until now. With the recent successful synthesis of ^13^C_6_-TATB (^13^C incorporated into the benzene ring), the same experiments have been used to show the source of the CO_2_ is the early breakdown of the TATB ring, not adventitious C from impurities and/or adsorbed CO_2_. A shift in mass m/z 44 (CO_2_) to m/z 45 is observed throughout the decomposition process indicating the isotopically labeled ^13^C ring breakdown occurs at the onset of thermal decomposition along with furazan formation. Partially labeled (N^18^O_2_)_3_-TATB confirms at least some of the oxygen comes from the nitro-groups. This finding has a significant bearing on decomposition computational models for prediction of energy release and deflagration to detonation transitions, with respect to conditions which currently do not recognize this oxidation step.

## Introduction

TATB is an important explosive compound because of extensive use in munitions. Typically formulated with a small percentage of polymer to modify properties, the material has been utilized world-wide in weapon systems. TATB is widely viewed as one of the most stable insensitive high explosives (IHE)s, as it is not easily detonated by external stimuli^1^. It does not undergo the thermal sequence of deflagration-to-detonation (DDT). It requires a proper detonation chain to initiate, so handling the material is relatively free from accidental initiation if proper safety methods are followed. One aspect of this safety envelope is how the material responds to temperature extremes; whether this material becomes more sensitive and is no longer safe to handle when subjected to abnormal thermal environments. This issue has been the subject of extensive research interest for close to 50 years^2,3^. The objective has been to understand the behavior experimentally to construct computer models predicting behavior for any thermal exposure condition^4–8^.

**Figure 1:**
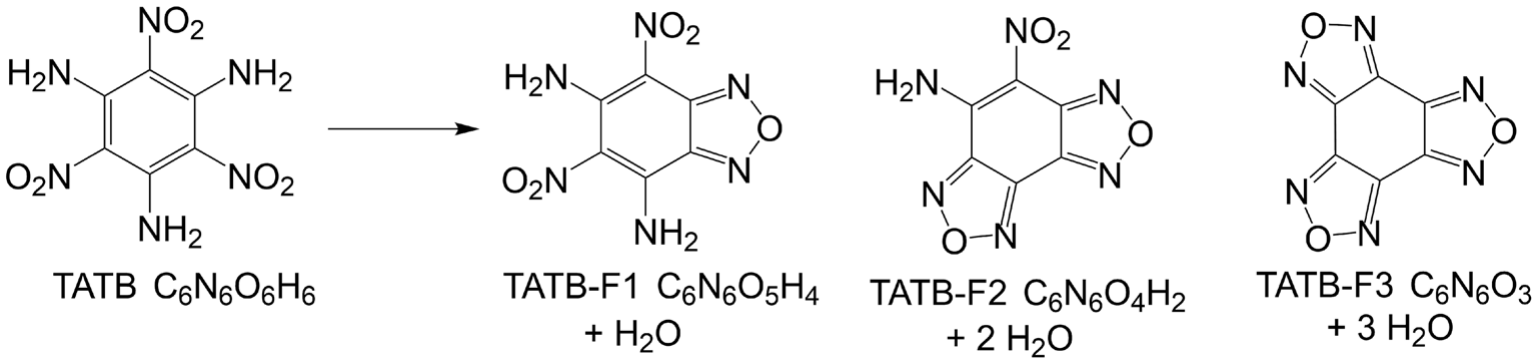
Furazan pathway for TATB decomposition resulting in the production of H_2_O via nitro-amine reactions (benzo-monofurazan (F1), benzo-difurazan (F2), benzo-trifurazan (F3)).

The consensus in the literature shows TATB decomposes thermally through the furazan reaction network shown in Fig. 1^9–12^. Several studies have indicated the first step in the decomposition is condensation of adjacent amino- and nitro-groups forming H_2_O and the furazan ring. Spectroscopic evidence shows this sequence probably proceeds stepwise until all substituents are condensed. Subsequently, the rings fall apart leading to light-gas formation, such as C_2_N_2_ and HNCO, HCN, etc^10–18^. The molecular profile of TATB as it relates to DDT transitions has traditionally been overlooked in the literature and our study highlights novel molecular pathways that occur as IHE is heated above its stability limit. These pathways can help constrain the physiochemical properties of current and future IHE compounds and allow prediction of the behavior and safe handling of energetic materials. This study has established a new understanding of IHE decomposition as it pertains to DDT transitions and lays the foundations for linking complex molecular processes to kinetic and thermodynamic measurements of IHE.

## Results

**Figure 2:**
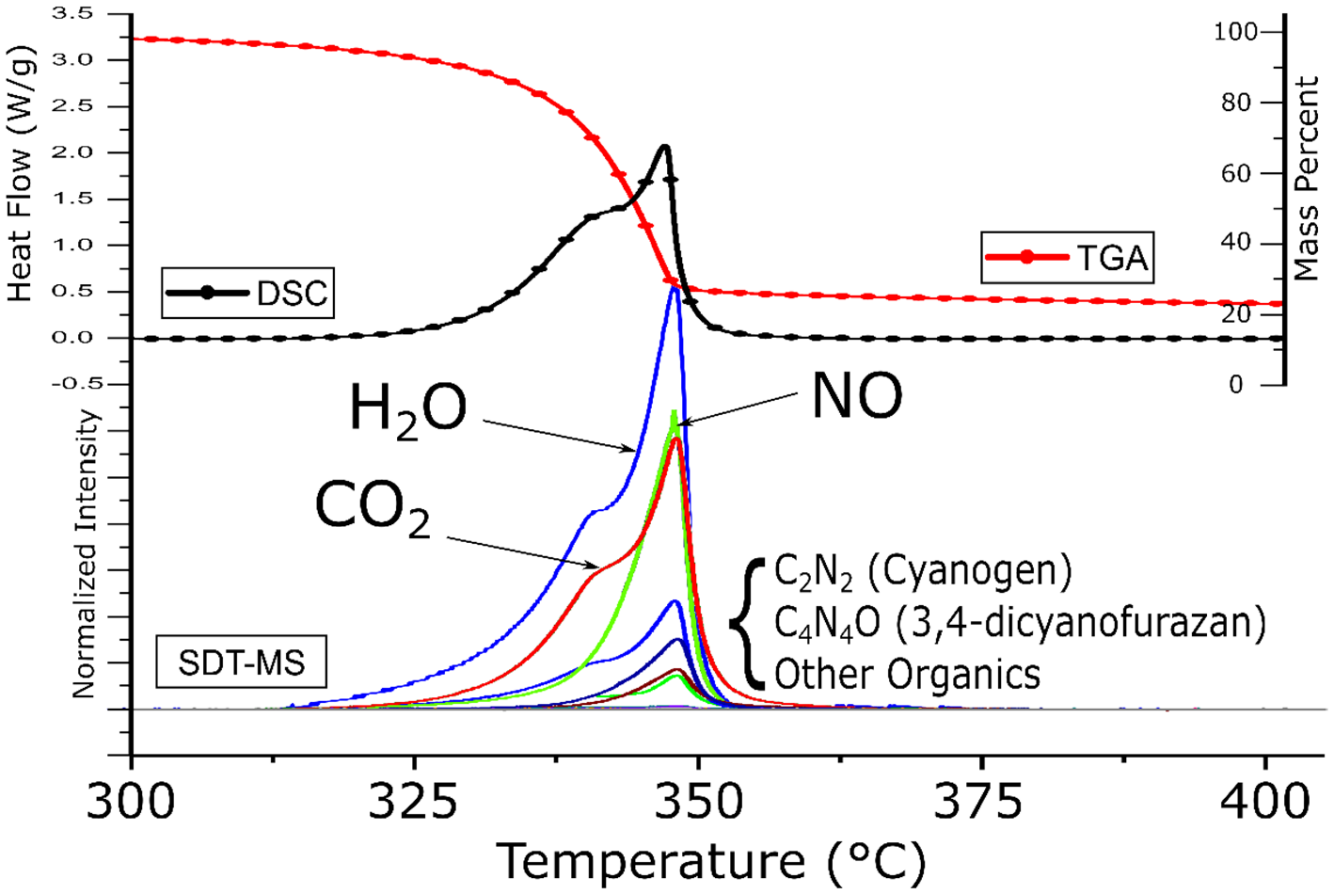
Thermal decomposition of TATB by SDT-MS, 1 °C/min heating rate: (top) heat flow and weight loss, (bottom) MS of gaseous effluent.

Figure 2 shows the thermal decomposition of TATB as measured by SDT-MS. The upper part of the figure shows the heat flow and weight loss as a function of temperature. The heat flow exhibits the major exotherm with a maximum at 347.1 °C (1 °C/min heating rate) with a smaller exotherm as a leading-edge shoulder. The weight loss profile shows most of the decomposition occurs during heat flow for these two exotherms. Using constant heating rate thermal decomposition methods, we have detected early evolving H_2_O, but in addition, early evolving CO_2_ as well. The bottom part of Fig. 2 shows the mass spectra for various light gases evolved during the decomposition. All the gases align with the sharp major exotherm, with some also aligning with the leading-edge shoulder. The blue trace shows early evolving H_2_O, consistent with the furazan decomposition route. However, the red trace shows early evolving CO_2_ which is not consistent with the furazan condensation and indicates an oxidation process is occurring. The carbon source of this oxidation is either adventitious carbon from absorbed species (impurities or CO_2_) or from the ring of the TATB. To understand this source, ^13^C_6_-TATB was synthesized and was further examined using pyrolysis GC-MS in evolved gas analysis (EGA) mode EGA-GC-MS.

**Figure 3:**
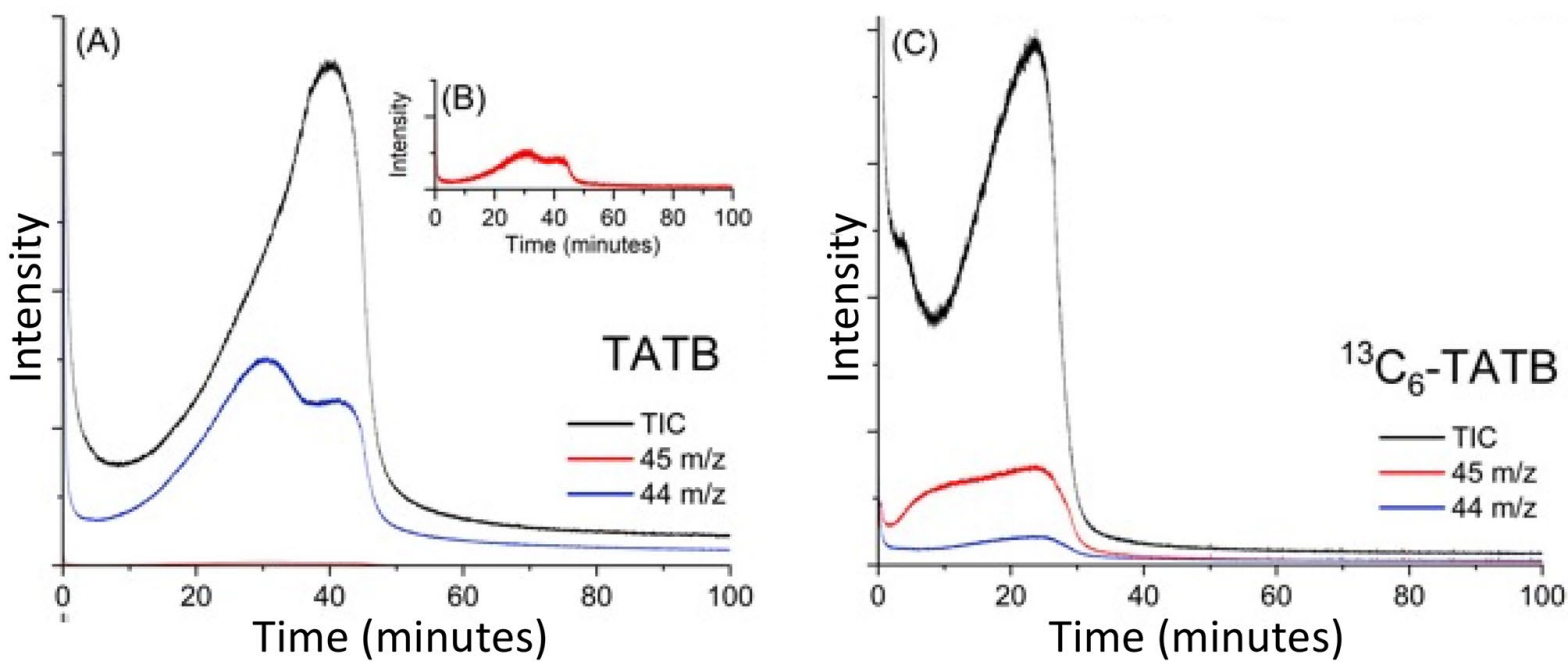
EGA-GC-MS of TATB (**A**), inset, m/z 45 scaled 500 X (**B**), and ^13^C_6_-TATB (**C**) at 330 °C: total ion current and extracted ion chromatograms for m/z 45 and 44 are shown with black, red, and blue lines, respectively.

Figure 3 shows the ion behavior with respect to time from the EGA-GC-MS analyses of the TATB and ^13^C_6_-TATB heated isothermally at 330 °C. The EGA profiles show the total ion chromatogram (TIC) for the unlabeled TATB and ^13^C_6_-TATB along with extracted ion chromatograms (EIC) for m/z 44 and 45. In both cases, there is an early intense pulse of gases occurring in the first 30 s of heating arising from minor impurities and instrument background. The bulk of gases evolving from TATB thermal decomposition start ~ 5–10 min later. For TATB, early evolving CO_2_, m/z 44, is evident; for ^13^C_6_-TATB, there is very little CO_2_, m/z 44, produced. The EICs for 45 m/z represent ^13^CO_2_ forming from oxidation of the TATB phenyl ring. For unlabeled-TATB there is almost no intensity for m/z 45 while for the ^13^C_6_-TATB, the profile shows m/z 45 ion evolving both early and late. The residual 44 m/z CO_2_ in the ^13^C_6_-TATB profile is due to 94 % isotopic purity^19^.

It is significant that the ^13^C material decomposes about twice as fast as ^12^C material. The faster decomposition was verified using thermal analysis (simultaneous mass loss and heat flow) at both isothermal and constant heating rate conditions using methods described elsewhere^20^. What drives the increased decomposition rate in the ^13^C_6_-TATB remains unknown, however it may be related more to changes in the TATB crystal structure and alignment of carbon and nitro groups to promote oxidation than an inverse kinetic isotope effect due to the minor mass defect between ^12^C and ^13^C. For heating rates of 0.5 to 10 °C/min, decomposition rate of ^13^C_6_-TATB maximizes about 6 °C lower^20^.

**Figure 4:**
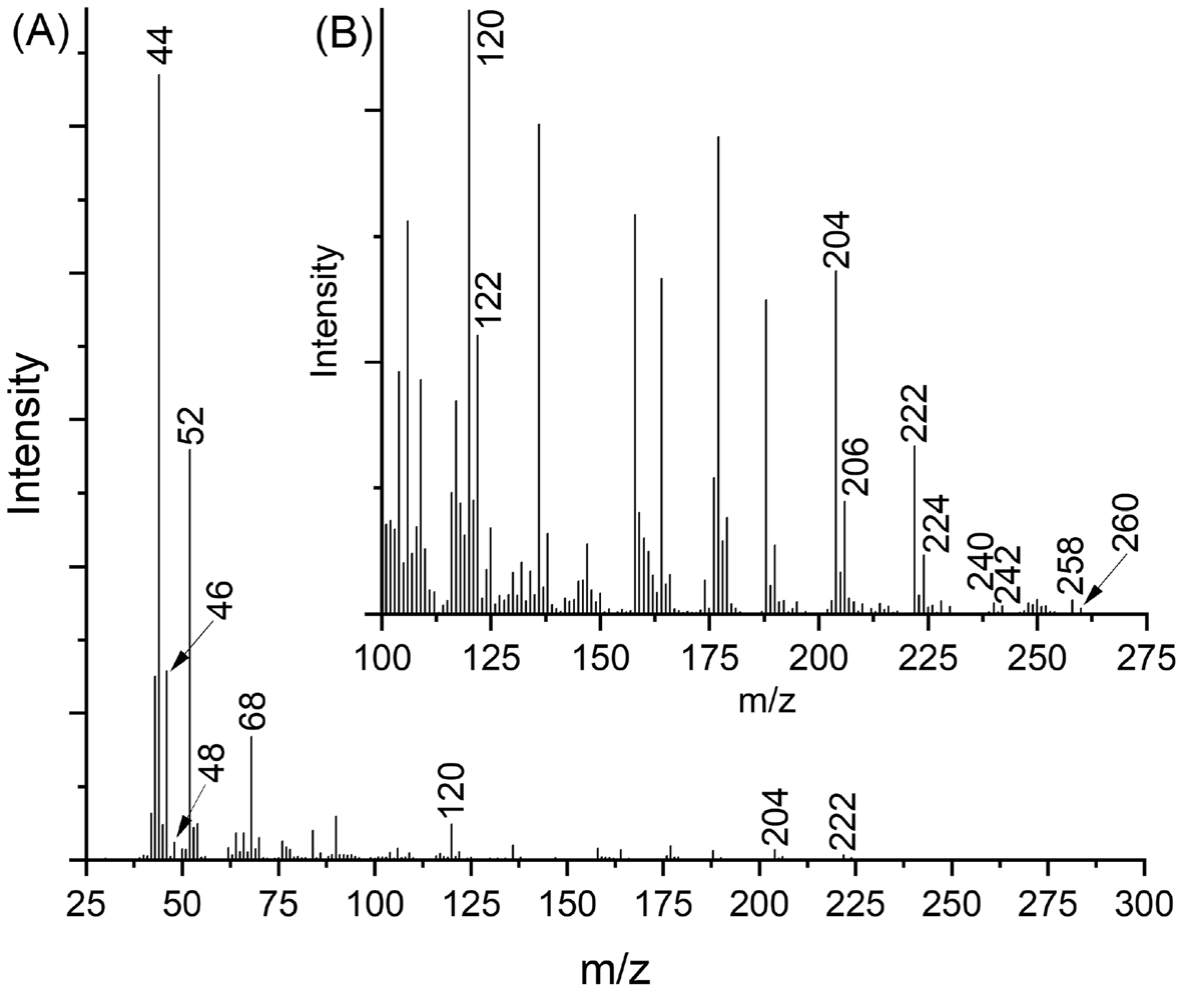
Mass spectral analyses of (N^18^O_2_)_3_-TATB isothermally treated at 330 °C. Scans added from 5 to 55 min; (**A**) full scan, (**B**) expanded parent ion region showing + 2 amu additions to TATB (m/z 258), F1 (m/z 240), F2 (m/z 222), F3 (m/z 204) and 3,4-dicyanofurazan (m/z 120).

An attempt to completely substitute ^18^O into the nitro-group on the TATB only was partially successful yielding less-than-complete ^18^O-substituted TATB. However, the substitution was successful enough to use analytically and demonstrate at least a partial source of the oxidation of the carbon. Figure 4A shows the EGA mass spectra from 5 to 55 min of the crude (N^18^O_2_)_3_-TATB also isothermally heated at 330 °C. For CO_2_, m/z 44 is the predominant feature in the low mass range. The ion m/z 46 is also evident from CO^18^O, which would occur with the only partially labeled material, indicating the early evolving CO_2_ is likely occurring due to involvement in the oxidation by nitro group on TATB. The ^18^O labeling mostly produced one ^18^O atom on the NO_2_ group of TATB. The peak at m/z 46 could also arise from NO_2_, however the EGA mass spectra from unlabeled TATB produces a minor peak at m/z 46. This is likely due to the high reactivity of NO gas, that is consumed in oxidation reactions. The incorporation of only one ^18^O into the CO_2_ molecule indicates it is likely a direct oxidation by NO_2_, either as a gas or neighboring molecule, transferring two O atoms to the C of the phenyl ring. Low intensity m/z 48 was observed in the (N^18^O_2_)_3_-TATB and likely formed from the small amount of double labeled NO_2_ present in the sample further supporting the strong oxidant as the driving force. The addition of ^18^O into the other major thermal decomposition products was observed as well. For example, as appears in Fig. 4B, TATB and the furazans all increased by 2 amu.

## Discussion

**Figure 5:**
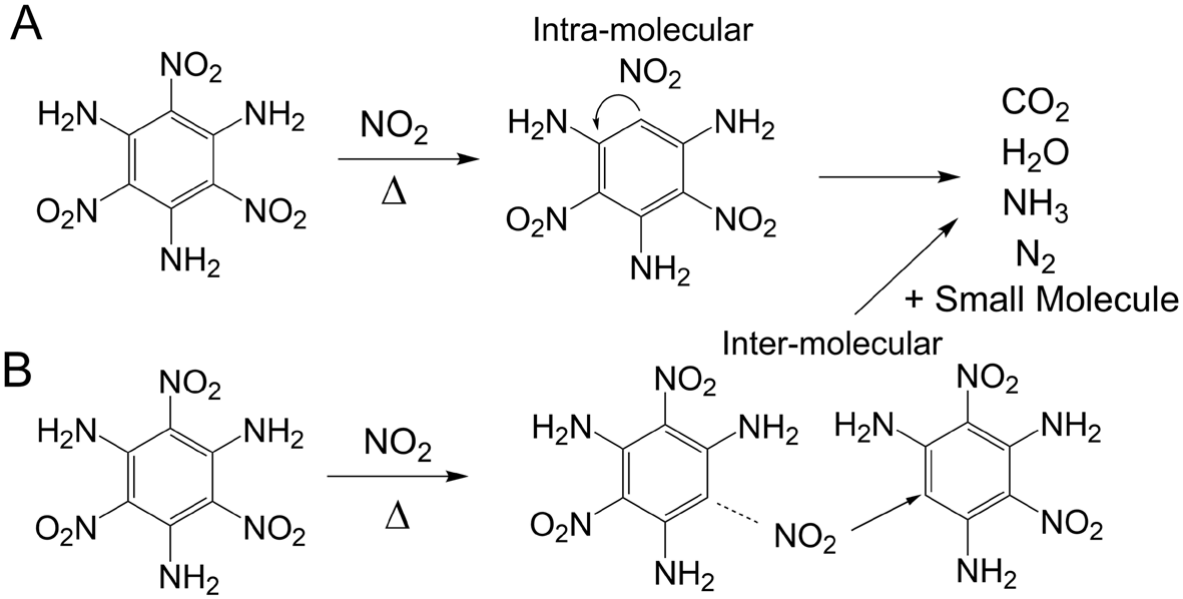
Phenyl ring carbon oxidation decomposition pathway for TATB driven by direct intra-molecular reactions or via inter-molecular reactions with NO_2_ from adjacent TATB molecules.

The formation of CO_2_ from the generation of NO_2_ was verified with the ^13^C and ^18^O labeled TATB experiments and support the auto-oxidation of TATB with heating. The extracted ion chromatograms from the EGA experiments with isothermal heating reveal rapid oxidation takes place when TATB is heated to decomposition temperatures. This occurs early in the isothermal experiments at 330 °C and the release of NO_2_ and CO_2_ gas coincide as a portion of the TATB is oxidized. However, the intensity in the mass spectrometer from NO_2_ gas is minor. This is likely due to instrument response and possibly because it is instantaneously consumed by redox reactions as TATB decomposes to CO_2_ and N_2_ gas. The nitro groups on TATB are predicted to be the weakest bond in the TATB structure^21,22^ and NO_2_ scission into the gas-phase is predicted at temperatures > 330 °C. This process occurs rapidly in the first min of isothermal heating at 330 °C. The formation of isocyanic acid (HNCO) would be favored in this initial oxidation event if NO_2_ gas is oxidizing the carbon ring and attaching oxygen to a carbon next to an amine group^22,23^. Nitrogen oxide (NO) gas is also an oxidant that may be playing a role in the auto-oxidation process, and Fig. 2 shows the verification of co-evolution by SDT-MS. In related ^15^N isotopically labeled experiments we were able to tentatively identify N_2_ as a breakdown product^16^. Other studies have verified the presence of N_2_ gas during thermal decomposition of TATB^13^. Figure 5 shows the auto-oxidation reaction. The NO_2_ in the graphic is meant to represent the start of the oxidation step, but not necessarily the active oxidant, as discussed above.

Much effort has been put into trying to understand how solid-phase-only reactions initiate. For TATB, kinetics and modelling studies suggest the first step is due to H_2_O formation, (probably through the well-established poly nitro decomposition process with gamma position hydrogens^24,25^) which would implicate the furazan reaction network shown Fig. 1. This has been consistent with analytical isolation of reaction products^9–12^. However, these new results show an oxidation reaction is occurring early and the source of the carbon is the TATB ring itself. This reaction has never been shown before for TATB, but NO_2_ involvement in oxidation at the onset of explosive decomposition (often leading to detonation) has often been implicated with nitro-only type explosives^26,27^. In the TATB case, the two reaction mechanisms, furazan formation and oxidation by NO_2_ appear to be in competition early on as a cascade of redox reactions occur. The formal oxidation numbers for the nitro and amino groups on the TATB are + 3 and − 3, respectively. If a furazan molecule/s/ forms, the oxidation number for both N atoms would have to shift to + 1. A series of oxidation/reduction reactions would have to occur for this to happen and represent an overlooked step in the thermal decomposition of TATB. The steps leading to this early oxidation, CO_2_ generation, dehydration and furazan formation remain unclear, and the generation of NO_2_ gas in addition to the inter/intra molecular electron shuttling could play a role. The TATB molecules are firmly locked into a planar array with other TATB molecules through interactions such as hydrogen bonding. These arrays are stacked in a tertiary structure, also stabilized by hydrogen bonding, attributed to the remarkable insensitivity of the TATB to external stimuli and hence the label as an IHE^28,29^. How the solid-state structure can reorganize to allow for this oxidation to occur is a matter for future examinations.

These results could also be critical for understanding why TATB (and formulations) do not go from deflagration to detonation (DDT), another feature desirable about IHE. Most monomolecular military type explosives have nitro groups as the functional substitution to produce explosive power. The amino group was introduced to provide better thermal stability^30,31^. That introduction could be the key to keeping TATB from DDT. Our research reveals this newly elucidated oxidation mechanism, which is proposed to drive explosives to detonations^26,27^, in the case of TATB, has competition from the furazan condensation reaction for available NO_2_. This is enough competition early on to keep the burning reaction from accelerating to create the wave front needed for detonation. Other, nitro-based only explosives do not have this competition for the oxidation source. These results lay the foundation for understanding auto-oxidation thermal decomposition in nitro amine based IHEs and a step towards the mechanistic understanding of what makes a robust IHE.

## Methods

### STD measurements

Constant heating rate: TA Instruments Q600 STD Simultaneous Differential Scanning Calorimetry-Thermogravimetric Analysis coupled with a Pfeiffer Vacuum Thermostar GSD 320 T3 Mass Spectrometry (SDT-MS); 1 °C/min heating rate; room temperature to 600 °C scan range; 3.0 g sample; alumina sample pan; 200 ms/amu, N_2_ carrier gas; selective ions monitored: m/z 12, 14, 16, 17, 18, 27, 28, 29, 30, 32, 43, 44, 45, 52, 53, 68, 69, 120, 122^17^. Isothermal: Mettler-Toledo model TGA/DSC 3+ with autosampler; 40-μL pans with crimped 50-μm pinhole lids sample size 1.9–3.8 mg of powder. As an example, isothermal treatment at ~ 332 °C of TATB gave maximum heat flow at 55 min; for ^13^C_6_-TATB maximum heat flow was at 35 min^20^.

### Synthesis

The ^13^C label was introduced by converting ^13^C labeled aniline to ^13^C trichloro benzene followed by wet amination synthesis to make ^13^C_6_-TATB (^13^C_6_H_6_N_6_O_6_), characterized by SS-NMR, FTIR, MS, DSC; 94% isotopically pure, 92 wt% chemically pure. The ^18^O labeled was introduced by the same synthesis method as in delineated elsewhere^19^ with modifications of trichloro benzene nitrated with KN^18^O_3_. Only partially substituted products were isolated due to exchange with the acids during synthesis—1 ^18^O-nitro group: 32.44%; 2 ^18^O-nitro groups: 7.87%; 3 ^18^O-nitro groups: 1.04%.

### Py-GC-MS

Pyrolysis GC-MS (Py-GC-MS) experiments were performed using an Agilent 7890B GC coupled to a 7010B MS/MS mass spectrometer and a Frontier Lab pyrolyzer (EGA/PY-3030D), auto-shot sampler (AS-2020E) and selective sampler (SS-2010E); a steel column (3 m × 150 µm inside diameter) was used to measure evolving gases with no chromatographic separation; silver foil was used generate pseudo-confined samples, comparable to the SDT-MS experiments with 50-µm pinholes; scan on the first quadrupole mass spectrometer from mass 29 to 650, using a 600 ms dwell time. Samples were heated isothermally at 330 °C for 100 min^32^.
